# NMR Profiling of North Macedonian and Bulgarian Honeys for Detection of Botanical and Geographical Origin

**DOI:** 10.3390/molecules25204687

**Published:** 2020-10-14

**Authors:** Dessislava Gerginova, Svetlana Simova, Milena Popova, Marina Stefova, Jasmina Petreska Stanoeva, Vassya Bankova

**Affiliations:** 1 Bulgarian NMR Centre, Institute of Organic Chemistry with Centre of Phytochemistry, Bulgarian Academy of Sciences, Acad. G. Bonchev str. Bl. 9, 1113 Sofia, Bulgaria; dpg@orgchm.bas.bg; 2 Laboratory Chemistry of Natural Products, Institute of Organic Chemistry with Centre of Phytochemistry, Bulgarian Academy of Sciences, Acad. G. Bonchev str. Bl. 9, 1113 Sofia, Bulgaria; popova@orgchm.bas.bg; 3 Institute of Chemistry, Faculty of Natural Sciences and Mathematics, Ss. Cyril and Methodius University, 1000 Skopje, North Macedonia; marinaiv@pmf.ukim.mk (M.S.); jasmina.petreska@pmf.ukim.mk (J.P.S.)

**Keywords:** NMR spectroscopy, honey, honeydew honey, geographical origin, classification

## Abstract

Bulgaria and North Macedonia have a long history of the production and use of honey; however, there is an obvious lack of systematic and in-depth research on honey from both countries. The oak honeydew honey is of particular interest, as it is highly valued by consumers because of its health benefits. The aim of this study was to characterize honeydew and floral honeys from Bulgaria and North Macedonia based on their NMR profiles. The 1D and 2D ^1^H and ^13^C-NMR spectra were measured of 16 North Macedonian and 22 Bulgarian honey samples. A total of 25 individual substances were identified, including quinovose, which was found for the first time in honey. Chemometric methods (PCA—principal component analysis, PLS-DA—partial least squares discriminant analysis, ANOVA—analysis of variance) were used to detect similarities and differences between samples, as well as to determine their botanical and geographical origin. Semiquantitative data on individual sugars and some other constituents were obtained, which allowed for the reliable classification of honey samples by botanical and geographical origin, based on chemometric approaches. The results enabled us to distinguish oak honeydew honey from other honey types, and to determine the country of origin. NMR was a rapid and convenient method, avoiding the need for other more time-consuming analytical techniques.

## 1. Introduction

Honey is consumed in large quantities all over the world, and its positive effect on human health has been known since ancient times. Bulgaria and North Macedonia have a long history of production and use of honey, dating back to the 1st Millennium BC. However, there is an obvious lack of systematic and in-depth research on Bulgarian and North Macedonian honey. The oak honeydew honey from both countries is of particular interest. Bees harvest honeydew honey from plant secretions produced as a result of an attack by insects sucking plant sap. Honeydew honey is highly valued by consumers because it is considered to be more beneficial to health than floral (nectar) honey [1]. Several studies have been published, demonstrating that the antibacterial and antioxidant activity of honeydew honey is superior to that of nectar honey [2]. Moreover, honeydew honey contains higher amounts of oligosaccharides than the floral one [3], and honey oligosaccharides have been found to possess potential prebiotic activity increasing the populations of beneficial bacteria in the human gut [4].

There are different approaches to the assessment of botanical and geographical origin of honey. Melissopalynological analysis can tell the main pollen type, the presence of honeydew elements, and the geographical origin through pollens specific to a certain area. However, pollen analysis is time consuming and requires expertise. In addition, pollen does not necessarily reveal the actual nectar source of the honey: some pollen types can be over- or under-represented in relation to nectar [5]. Together with pollen analysis, combinations of physicochemical and chemical parameters are used in the quality assessment and characterization of honeys [6]. On the other hand, Nuclear Magnetic Resonance spectroscopy provides a simple method to obtain global information about complex samples, making it ideal for applications in honey research [7]. This study was undertaken to characterize honeydew and floral honeys from Bulgaria and North Macedonia based on their NMR profiles. Chemometric methods were used to detect similarities and differences between samples, as well as to determine their botanical and geographical origin. The study of the chemical composition of honey in both countries will make it possible to produce well-characterized honeys for the international market.

## 2. Results and Discussion

Honey consists essentially of different sugars; honey carbohydrates are made up of about 70% monosaccharides (mainly glucose and fructose), 10–15% disaccharide, and small amounts of tri- and tetrasaccharides [8]. Thus, sugar profiles are important characteristics of different types of honey. In the last years, NMR has been successfully applied to authenticate honey samples in relation to geographical or botanical origins based on their sugar profiles [9,10].

### 2.1. NMR Analysis and Identification of Honey Constituents

Sixteen North Macedonian and twenty-two Bulgarian honey samples were studied, obtained from local beekeepers and/or commercial producers. ^1^H and ^13^C-NMR spectra were recorded for each sample. Some of the samples were declared as honeydew honeys, others as “forest”, “meadow”, or polyfloral honeys. The first goal of this study was to discriminate oak honeydew honey from all other honeys. To distinguish unambiguously the oak honeydew honeys, a simple NMR approach developed earlier was applied. This approach is based on the occurrence of the signals of the methylene group of the deoxyinositol quercitol in the ^1^H and ^13^C-NMR spectra of honey [11]. Quercitol is regarded as a good taxonomic marker for the genus *Quercus* [12]. The presence of noticeable amounts of quercitol (over 0.25%) proved the identity of 10 Bulgarian and 7 Macedonian samples as oak honeydew honey.

Based on 1D and 2D ^1^H and ^13^C-NMR spectra, 25 individual substances were identified: sugars, amino acids, organic acids, alcohols, 5-hydroxymethylfurfural, etc. (Table 1). Numerous overlapping multiplets in the anomeric spectral region of the honey proton spectra prevent reliable quantification by ^1^H-NMR. For this reason, semiquantitative analysis was performed based on the intensity of the ^13^C-NMR signals using 3 monosaccharides (glucose, fructose, quinovose), 13 disaccharides (sucrose, kojibiose, α,α- and α,β-trehalose, trehalulose, maltose, isomaltose, maltulose, isomaltulose, nigerose, leucrose, turanose, gentiobiose), 5 trisaccharides (raffinose, melezitose, 1-kestose, panose, erlose), proline, quercitol, and 2,3-butanediol. Included in the analysis are also the ^13^C signal intensities of 16 unidentified compounds that proved to be important for the discrimination of the different honey types. Concentration range (min-max) and average content (avg) of studied components are represented in Table S2.

**Figure 1:**
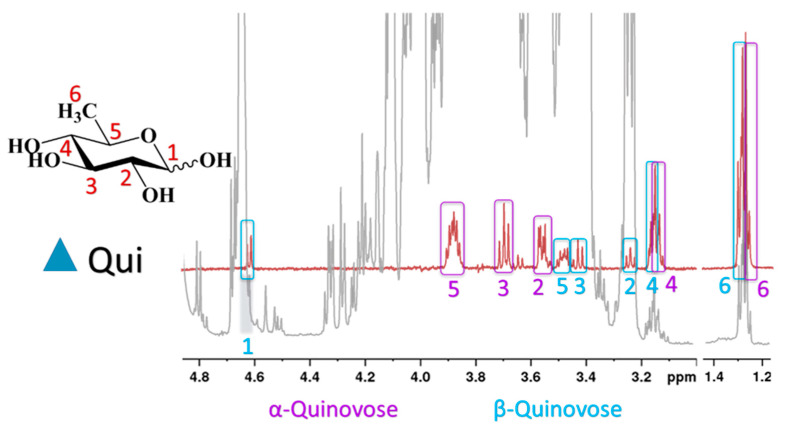
Selective TOCSY spectrum of quinovose (red line) and ^1^H spectrum of honey (grey line).

Interestingly, the reducing monosaccharide quinovose (6-deoxyglucose) was detected in higher quantities in some Nord Macedonian honeys. It was identified by a selective TOCSY (TOtal Correlated SpectroscopY) experiment for sample M11 (Figure 1), and it should be noted that its presence in honey has not been described so far.

Quinovose has been found in the latex of *Ipomoea carnea* [13], and was identified as a part of the sugar moiety of hydroxy fatty acid glycosides isolated from plants of the family Convolvulaceae [14], and of triterpene glycosides found in marine organisms [15]. The presence of the reducing sugar quinovose was detected in 7 of the 16 samples of North Macedonian honey, and in 6 of the 22 Bulgarian samples. The origin of this monosaccharide in honey samples remains unclear.

### 2.2. Chemometric Analysis

**Figure 2:**
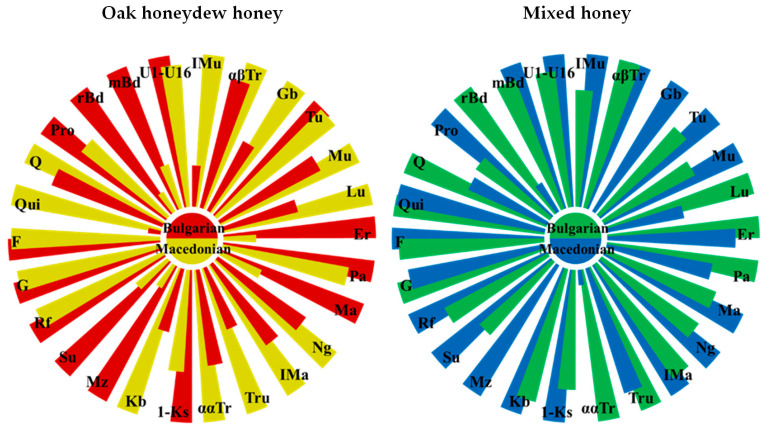
Nightingale’s Rose Diagrams of the content of 26 constituents in Bulgarian and North Macedonian honeys (Qui—quinovose, F—fructose, G—glucose, Rf—raffinose, Su—sucrose, Mz—melezitose, Kb—kojibiose, 1-Ks—1-kestose, ααTr—α,α-trehalose, Tru—trehalulose, IMa—isomaltose, Ng—nigerose, Ma—maltose, Pa—panose, Er—erlose, Lu—leucrose, Mu—maltulose, Tu—turanose, Gb—gentiobiose, αβTr—α,β-trehalose, IMu—isomaltulose, U1–U16—sum of 16 unidentified constituents, Q—quercitol, mBd—meso-2,3-butanediol, rBD—rac-2,3-butanediol, Pro—proline).

Comparison between North Macedonian and Bulgarian oak honeydew honey samples revealed a number of similarities and differences. The presence of noticeable amounts of quercitol is characteristic for the oak honeydew honey from both countries. However, Macedonian honeydew honeys differ from the Bulgarian ones in the amounts of most of the identified constituents. Graphically, these differences are depicted by the Nightingale’s Rose Diagrams (Figure 2). While the amounts of glucose, fructose, raffinose, panose, and turanose are nearly constant, larger quantities of 1-kestose, melezitose, erlose, maltose, proline, and 2,3-butanediol, and markedly smaller amounts of kojibiose, trehalulose, leucrose, gentiobiose and isomaltulose are present in the Bulgarian oak honeydew honey.

**Figure 3:**
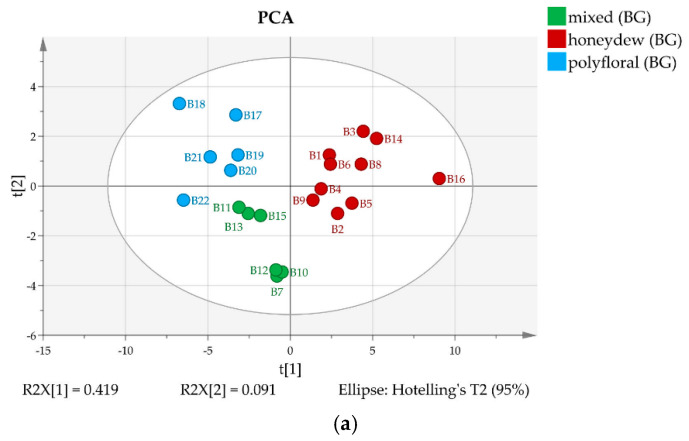

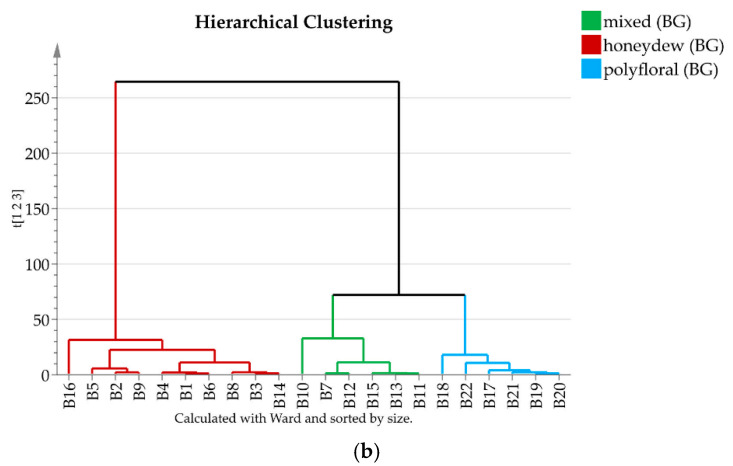
(**a**) 2D score PCA plot of Bulgarian honey samples using quantities of all components; (**b**) Dendrogram of Bulgarian honey samples.

**Figure 4:**
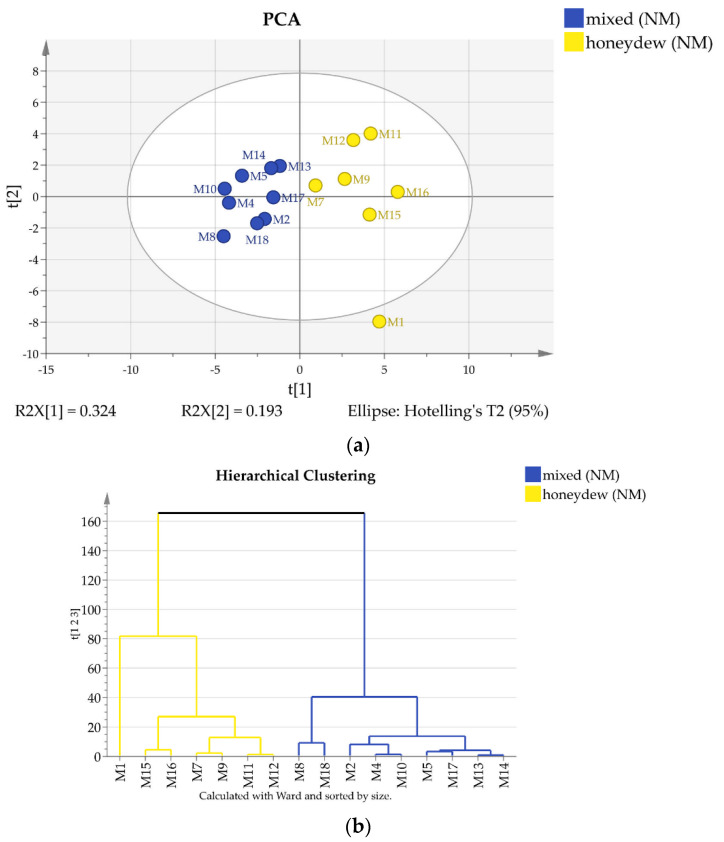
(**a**) 2D score PCA plot of North Macedonian honey samples using quantities of all components; (**b**) Dendrogram of North Macedonian honey samples.

The semiquantitative ^13^C-NMR data were subjected to chemometric analysis. Based on the ^13^C-NMR signal intensities Principal Component Analysis and Hierarchical Clustering were performed separately for Bulgarian and for North Macedonian honey types. The results are displayed in Figure 3 and Figure 4, respectively.

The 22 Bulgarian samples are clearly separated by both methods in three groups that can be ascribed as polyfloral, honeydew, and mixed (polyfloral and honeydew) type (Figure 3), while both clustering and PCA indicate only two groups for the measured North Macedonian samples. It turned out that some of the samples (B11, B15, M5, M12 and M17) contain trehalulose instead of quercitol as an additional honeydew marker and can be classified as mixed or honeydew [16].

**Figure 5:**
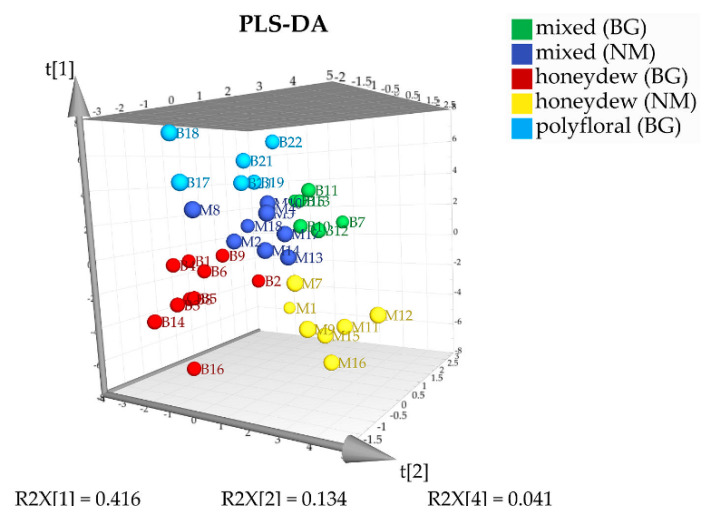
PLS-DA 3D score plot of the honey samples.

In order to confirm the proposed classification for geographical and botanical origin of the analyzed samples, we used supervised chemometric methods—analysis of variances (ANOVA) and partial least squares discriminant analysis (PLS-DA). ANOVA with statistical certainty of 90% (F_crit_ = 2.12) was used for determination of the statistically significant components that allowed us to distinguish between three types of honeys from Bulgaria and North Macedonia—mixed, honeydew, and polyfloral honeys. Thirty-one substances (sugars, unidentified components, quercitol, and 2,3-butanediol) were selected based on their F criteria (Table 1) to give a stable model. Semiquantitative data for these 31 substances were used in the partial least squares discriminant analysis. Five PLS components explain 71.6% of the variation (R2X(cum) = 0.716; R2Y(cum) = 0.677; Q2(cum) = 0.347). In the 3D score plot on Figure 5 the first, the second and the fourth components are visualized. The graph clearly demonstrates that PLS-DA allows reliable classification of the samples both by botanical and geographical origin. Validation of the PLS-DA model was performed using a test with 25 permutations and receiver operating characteristic (ROC) curve analysis (Figures S1–S6).

**Figure 6:**
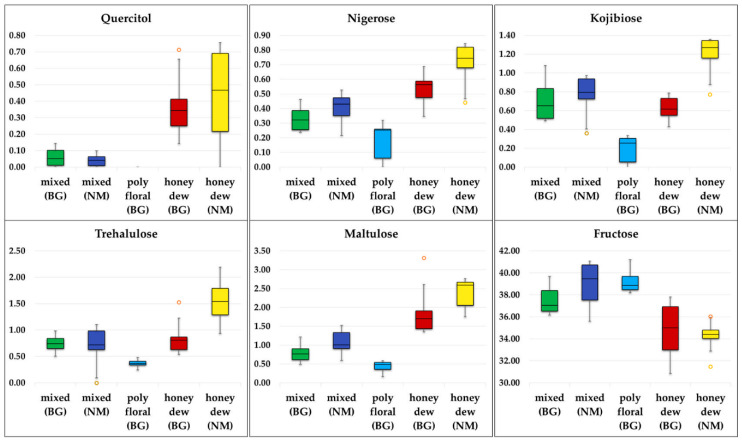
Box plot presentation for quercitol, nigerose, kojibiose, trehalulose, maltulose and fructose.

Several box plots for the individual components of the five honey types indicate good differentiation of the geographical or the botanical origin, or both (Figure 6).

Higher contents of quercitol and nigerose are typical for honeydew honeys in general. In the group of honeydew honeys, North Macedonian samples contain higher amounts of kojibiose, maltulose, nigerose, trehalulose, and quercitol, while the Bulgarian samples were characterized by a higher concentration of erlose, maltose, and proline. In the group of mixed honeys, the differences are smaller, Bulgarian mixed samples contain less fructose and maltulose, and no melezitose. Quercitol, melezitose, and U11 cannot be found in polyfloral honeys (there are only Bulgarian samples).

The validity of the PLS-DA model used could also be proved by the determined misclassification table (Table 2). Thirty-seven of the 38 samples (97.37%) were correctly classified. Only one mixed North Macedonian honey sample (M18) was predicted as Bulgarian honeydew honey. The results demonstrate that the model using ^13^C-NMR profiling information for 31 substances is able to reliably predict the botanical and geographical origin of the investigated honey types.

## 3. Materials and Methods

### 3.1. Honey Samples

A total of 38 honey samples (16 from North Macedonia and 22 from Bulgaria) with different botanical origin were analyzed using NMR spectroscopy (Table S1).

### 3.2. Sample Preparation

In total, 320 mg of each honey were diluted in 418 µL distillated water and 187 µL deuterated phosphate buffer solution, containing 5.8 mM deuterated trimethylsilylpropionic acid sodium salt (TSP). The pH of the samples was adjusted to 4.20 ± 0.02 with 8.5% H_3_PO_4_.

### 3.3. NMR Spectroscopy

The ^1^H and ^13^C-NMR spectra were recorded on a Bruker Avance II+ 600 spectrometer (Biospin GmbH, Rheinstetten, Germany) at 300.0 ± 0.1 K. 1H spectra with water suppression (noesypr1d pulse sequence) were acquired using 256 scans, 16 dummy scans, 64K data points, acquisition time of 5.15 s, and relaxation delay of 2.00 s. The parameters used in ^13^C spectra were pulse sequence zgdc30, pulse width 30° spectral width 238 ppm, 64 K data points, 8 K scans, acquisition time 0.90 s, and relaxation delay 1.05 s. The signal of α-fructofuranose at 104.34 ppm was used as internal reference corresponding to −2.82 ppm for the ^13^C TSP signal. Unambiguous assignment of the signals was achieved on the bases of 2D JRES, COSY, TOCSY, and HSQC spectra of several individual saccharides and honey samples (see Supplementary: page S2, Figures S7 and S8).

### 3.4. Semiquantitative Analysis

The ^13^C-NMR signals in the region 106–67 ppm were used for semiquantitative analysis. Only one non overlapped CH signal was selected for each saccharide, taking into account the molar mass, tautomeric form and its percentage, determined from the ^13^C-NMR spectra of the individual compounds in D_2_O. From the intensities of these signals the quantity of the corresponding compound was calculated (Table S1). Molar masses of the unidentified substances were determined using diffusion NMR experiment (DOSY). Their diffusion coefficients were between coefficients of mono- and disaccharides, thus molar mass equal to 200 g/mol was used for all of them. The calculated values of all components were summarized, and the quantity of each substance was expressed as a percentage. The masses of all compounds were calculated in g/100 g, taking into account a firm value of 20% for the moisture content in honey.

### 3.5. Multivariate Data Analysis

Multivariate analysis (PCA—principal component analysis, HCA—hierarchical clustering and PLS-DA—partial least squares discriminant analysis), ANOVA (analysis of variance) and Nightingale’s diagram were performed based on the semiquantitative ^13^C-NMR data [17,18]. The applied chemometric methods were accomplished using Excel [19] and Simca15 [20]. Permutation test and ROC (receiver operating characteristic) curve analysis were performed for validation of the PLS-DA model. The permutation plots are shown on Figures S1–S5. All real values of R2 and Q2 are higher than the permutated values that is a good indication of the predictive capability and validity of the PLS-DA model used. The excellent distinguishing between the classes is also proven by the receiver operating characteristic (ROC) plot shown on Figure S6. The areas under the ROC curve (Area Under Curve, AUC) of honeydew and polyfloral classes are equal to one and the AUC of mixed (BG) and mixed (NM) classes are very close to one, 0.98 and 0.97, respectively, demonstrating high quality of the classification model.

## 4. Conclusions

NMR profiling was successfully applied for characterization of Bulgarian and North Macedonian honeys. Semiquantitative ^13^C-NMR data on individual sugars and some other constituents allowed reliable classification of honey samples by botanical and geographical origin, based on chemometric approaches. The results enable the distinguishing of oak honeydew honey from polyfloral honey as well as for distinguishing both of them from mixed (polyfloral and honeydew) honey. The close geographical neighbors Bulgaria and North Macedonia obviously have different plant varieties; this makes it possible to determine the country of origin of honeys. The results allow to differentiate oak honeydew honey from other honey types, and to determine the country of origin. NMR was used as a rapid and convenient method, avoiding the need for other more time-consuming analytical techniques, such as pollen analysis and Isotope Ratio Mass Spectrometry.

## Figures and Tables

**Table 1:** Chemical shifts of used signals and their F* values.

^13^C δ [ppm]	Components	F (α = 0.1, F _crit_ = 2.12)
**Monosaccharides**		
**67.54**	Fructose (F)	12.89
**74.10**	Glucose (G)	4.32
**97.83**	Quinovose (Qui)	1.19
**Disaccharides**		
**76.36**	Sucrose (Su)	1.10
**89.30**	Kojibiose (Kb)	25.59
**93.00**	αα-Trehalose (ααTr)	1.75
**97.69**	Trehalulose (Tru)	12.68
**97.73**	Isomaltose (IMa)	11.32
**98.80**	Nigerose (Ng)	21.51
**99.48**	Maltose (Ma)	5.84
**100.04**	Leucrose (Lu)	7.53
**100.28**	Maltulose (Mu)	24.93
**100.65**	Turanose (Tu)	19.24
**102.45**	Gentiobiose (Gb)	0.61
**102.69**	α,β-Trehalose (αβTr)	15.56
**104.65**	Isomaltulose (IMu)	5.50
**Trisaccharides**		
**76.24**	Raffinose (Rf)	13.69
**83.36**	Melezitose (Mz)	3.36
**92.28**	Isokestose (1-Ks)	8.94
**99.56**	Panose (Pa)	5.67
**99.59**	Erlose (Er)	2.61
**Other compounds**		
**16.68**	Meso 2,3-butanediol (mBd)	7.62
**17.76**	Racemic 2,3-butanediol (rBd)	8.51
**23.65**	Proline (Pro)	1.69
**33.22**	Quercitol (Q)	11.83
**Unidentified compounds**		
**11.93**	U16	2.74
**96.71**	U15	1.03
**97.79**	U14	1.17
**98.16**	U13	26.62
**99.16**	U12	0.89
**100.76**	U11	14.92
**101.23**	U10	2.80
**101.70**	U9	2.53
**102.30**	U8	1.02
**102.77**	U7	3.27
**103.31**	U6	2.43
**103.38**	U5	1.63
**103.42**	U4	4.09
**103.49**	U3	5.34
**103.59**	U2	5.50
**104.11**	U1	10.37

* F value indicates the variable is statistically significant for the discrimination model.

**Table 2:** Misclassification table of mixed, honeydew (hdew), and polyfloral honeys the for PLS-DA model used (SIMCA15).

	Predicted	Members	Correct	Mixed (BG)	Mixed (NM)	Hdew (BG)	Hdew (NM)	Polyfloral (BG)	No Class
Actual									
**mixed (BG)**		6	100%	6	0	0	0	0	0
**mixed (NM)**		9	88.89%	0	8	1	0	0	0
**hdew (BG)**		10	100%	0	0	10	0	0	0
**hdew (NM)**		7	100%	0	0	0	7	0	0
**polyfloral (BG)**		6	100%	0	0	0	0	6	0
**No class**		0		0	0	0	0	0	0
**Total**		38	97.37%	6	8	11	7	6	0
**Fisher’s exact test**		1.40 × 10^−22^							

## Supplementary Materials

The following are available online. Supplementary file 1: Assignment procedures, Table S1: Honey samples used for NMR analysis, Table S2: Concentration range (min-max) and average content (avg) of studied components according to origin of honey, Figure S1: Permutation test for Bulgarian mixed honey class in PLS-DA model, Figure S2: Permutation test for North Macedonian mixed honey class in PLS-DA model, Figure S3: Permutation test for Bulgarian honeydew honey class in PLS-DA model, Figure S4: Permutation test for North Macedonian honeydew honey class in PLS-DA model, Figure S5: Permutation test for Bulgarian polyfloral honey class in PLS-DA model, Figure S6: ROC analysis for Bulgarian mixed, North Macedonian mixed, Bulgarian honeydew, North Macedonian honeydew and Bulgarian polyfloral honeys, Figure S7: Full ^1^H-NMR spectrum of sample M13 and assignment of the identified signals in the regions 1.10–2.40 ppm and 4.90–5.45 ppm, Figure S8: Full ^13^C-NMR spectrum of sample M13 and assignment of the signals, used in the chemometric analysis shown in Table 1.
