# Supplementary material for: NMR Profiling of North Macedonian and Bulgarian Honeys for Detection of Botanical and Geographical Origin
Source: Molecules. 2020 Oct 14;25(20):4687. doi: 10.3390/molecules25204687 (PMC7587359; doi:10.3390/molecules25204687)
Supplement: Supplementary file 1 [file molecules-25-04687-s001.pdf]

Supporting Information

for

NMR Profiling of North Macedonian and Bulgarian Honeys for Detection of Botanical and Geographical Origin

Dessislava Gerginova [1], Svetlana Simova [1,*], Milena Popova [2], Marina Stefova [3], Jasmina Petreska Stanoeva [3] and Vassya Bankova [2,*]

[1] Bulgarian NMR Centre, Institute of Organic Chemistry with Centre of Phytochemistry, Bulgarian Academy of Sciences, Acad. G. Bonchev str. Bl. 9, 1113 Sofia, Bulgaria; dpg@orgchm.bas.bg
[2] Laboratory Chemistry of Natural Products, Institute of Organic Chemistry with Centre of Phytochemistry, Bulgarian Academy of Sciences, Acad. G. Bonchev str. Bl. 9, 1113 Sofia; Bulgaria; popova@orgchm.bas.bg
[3] Institute of Chemistry, Faculty of Natural Sciences and Mathematics, Ss. Cyril and Methodius University, Skopje 1000, Republic of North Macedonia; marinaiv@pmf.ukim.mk (M.S.); jasmina.petreska@pmf.ukim.mk (J.P.S.)
* Correspondence: sds@orgchm.bas.bg (S.S.); bankova@orgchm.bas.bg (V.B.)

Assignment procedures

Full set of 1D and 2D NMR spectra of 6 mono-, 9 di- and 7 trisaccharides in D_2O at 300K have been acquired and used as reference for the honey samples. Use of sensitivity-enhanced HSQC spectra was usually sufficient for the assignment of the complementary ^1H/ ^{13}C NMR signals. In cases of severe overlapping additional techniques have been used for unambiguous assignment and check of the results. COSY spectra allow identifying coupled protons, while JRES spectra are useful for distinguishing between closely spaced proton signals with different multiplicities. 1D TOCSY (as shown in Fig. 1) have been used to assign complete spin systems to differentiate the overlapping signals belonging to individual stereoisomeric units (open chain, pyranoses, furanoses, alpha, beta). All signal assignments for the anomeric region are in agreement with the data, reported in the work of Petersen, B. O.; Hindsgaul, O.; Meier, S., "Profiling of carbohydrate mixtures at unprecedented resolution using high-precision ^1H-^{13}C chemical shift measurements and a reference library" in *Analyst* **2014**, *139*, 401-406 with a systematic difference of minus one ppm for the ^{13}C signals. As an illustration the ^1H and ^{13}C NMR spectra with the assignment of the identified signals are presented in Figures S7 and S8.

Table S1. Honey samples used for NMR analysis

Sample	Location of origin	Supplier	Declared botanical origin
	**North Macedonia**		
M1	Brod	beekeeper	polyfloral
M2	Kriva Palanka	beekeeper	polyfloral mountain
M4	Kruševo	beekeeper	polyfloral
M5	Debar	beekeeper	polyfloral forest
M7	Krushe	beekeeper	honeydew
M8	Petrovec	beekeeper	polyfloral meadow
M9	Rastesh	beekeeper	honeydew
M10	Orovnik	beekeeper	polyfloral forest
M11	Kriva Palanka	beekeeper	polyfloral meadow
M12	Trpejca	beekeeper	polyfloral meadow
M13	Mešeišta	beekeeper	acacia (black locust)
M14	Dojran	beekeeper	polyfloral meadow
M15	Brod	beekeeper	honeydew
M16	Brod1	beekeeper	honeydew
M17	Negotino	beekeeper	polyfloral
M18	Petrovec	beekeeper	polyfloral
	**Bulgaria**		
B1	Korkina	beekeeper	honeydew
B2	Ludogorie	commercial	honeydew
B3	Stara Zagora	commercial	honeydew
B4	Popina	beekeeper	honeydew
B5	Kalofer	commercial	honeydew
B6	Stara Zagora	commercial	honeydew
B7	Kostinbrod	beekeeper	honeydew
B8	Stara Zagora	commercial	honeydew
B9	Grozdiovo, Dolni Chiflik	commercial	honeydew
B10	Bardo, Ihtiman	beekeeper	forest
B11	Zhelyava	beekeeper	forest
B12	Padesh, Blagoevgrad	beekeeper	polyfloral forest
B13	Kraevo	beekeeper	forest
B14	Strandzha	commercial	honeydew
B15	NA*	commercial	polyfloral forest
B16	Plovdiv	commercial	honeydew
B17	Krushovitsa, Vratsa	beekeeper	polyfloral
B18	Yambol	beekeeper	polyfloral
B19	Korkina	beekeeper	polyfloral
B20	Rogosh, Plovdiv	beekeeper	polyfloral
B21	Dragoyna, Rhodopes	beekeeper	polyfloral
B22	NA*	commercial	polyfloral

* NA – not declared

Table S2. Concentration range (min-max) and average content (avg) of studied components according to origin of honey

class	***mixed (BG)***			***mixed (NM)***			***honeydew (BG)***			***honeydew (NM)***			***polyfloral (BG)***		
**g/100g**	**min**	**max**	**avg**	**min**	**max**	**avg**	**min**	**max**	**avg**	**min**	**max**	**avg**	**min**	**max**	**avg**
**IMu**	0.00	0.42	0.13	0.00	0.52	0.17	0.00	0.68	0.23	0.27	0.75	0.57	0.00	0.52	0.13
**αβTr**	0.14	0.40	0.24	0.11	0.30	0.24	0.22	0.49	0.37	0.31	0.48	0.41	0.00	0.20	0.13
**Gb**	0.00	0.00	0.00	0.00	0.20	0.05	0.00	0.24	0.05	0.00	0.30	0.08	0.00	0.21	0.06
**Tu**	0.84	1.48	1.23	0.88	2.08	1.52	1.43	2.09	1.86	1.55	2.15	1.84	0.24	1.07	0.74
**Mu**	0.49	1.21	0.79	0.59	1.53	1.08	1.36	3.31	1.87	1.75	2.77	2.37	0.17	0.59	0.43
**Lu**	0.04	0.50	0.19	0.04	0.20	0.11	0.14	0.37	0.21	0.22	0.72	0.35	0.00	0.09	0.05
**Er**	0.00	0.92	0.36	0.00	0.56	0.31	0.00	0.78	0.32	0.00	0.24	0.11	0.00	0.31	0.09
**Pa**	0.07	0.38	0.25	0.00	0.35	0.19	0.00	0.42	0.30	0.00	0.37	0.26	0.00	0.17	0.03
**Ma**	0.27	1.53	0.81	0.60	1.52	1.00	0.61	1.87	1.24	0.37	0.62	0.52	0.72	1.68	1.22
**Ng**	0.24	0.46	0.33	0.21	0.53	0.41	0.35	0.69	0.54	0.44	0.84	0.72	0.00	0.32	0.18
**IMa**	0.50	1.17	0.82	0.44	1.34	0.89	0.00	2.95	1.43	1.58	2.54	2.02	0.00	0.81	0.53
**Tru**	0.50	0.98	0.74	0.00	1.11	0.66	0.54	1.53	0.82	0.93	2.19	1.55	0.25	0.48	0.37
**ααTr**	0.00	0.78	0.20	0.00	0.30	0.05	0.00	0.22	0.11	0.00	0.44	0.15	0.00	0.00	0.00
**1-Ks**	0.00	0.25	0.10	0.00	0.29	0.13	0.18	0.62	0.40	0.00	0.53	0.29	0.00	0.16	0.03
**Kb**	0.49	1.08	0.71	0.36	0.97	0.77	0.43	0.79	0.63	0.77	1.36	1.20	0.00	0.34	0.19
**Mz**	0.00	0.00	0.00	0.00	0.21	0.06	0.00	0.24	0.10	0.00	0.14	0.03	0.00	0.00	0.00
**Su**	0.05	0.38	0.16	0.02	0.82	0.22	0.00	1.04	0.31	0.05	0.26	0.12	0.09	0.43	0.21
**Rf**	0.18	0.25	0.22	0.18	0.37	0.27	0.34	0.90	0.49	0.14	0.57	0.46	0.00	0.23	0.08
**G**	30.85	36.45	33.77	27.32	36.24	31.33	27.18	36.31	31.13	27.90	33.80	30.13	33.36	38.81	35.17
**F**	36.18	39.68	37.52	35.62	41.08	39.00	30.86	37.80	34.84	31.48	36.02	34.23	38.19	41.22	39.24
**Q**	0.00	0.14	0.06	0.00	0.10	0.04	0.14	0.71	0.35	0.00	0.76	0.43	0.00	0.00	0.00
**Pro**	0.04	0.09	0.06	0.04	0.17	0.09	0.08	0.15	0.11	0.06	0.14	0.08	0.02	0.17	0.10
**rBd**	0.00	0.02	0.01	0.00	0.01	0.00	0.02	0.15	0.06	0.00	0.10	0.02	0.00	0.00	0.00
**mBd**	0.00	0.06	0.02	0.00	0.05	0.02	0.04	0.30	0.13	0.00	0.23	0.05	0.00	0.00	0.00
**U1**	0.00	0.05	0.03	0.00	0.06	0.02	0.03	0.18	0.10	0.05	0.24	0.09	0.00	0.00	0.00
**U2**	0.02	0.06	0.04	0.00	0.18	0.03	0.10	0.23	0.15	0.00	0.33	0.05	0.00	0.05	0.02
**U3**	0.00	0.16	0.06	0.00	0.28	0.03	0.00	0.32	0.19	0.00	0.23	0.07	0.00	0.19	0.11
**U4**	0.00	0.20	0.06	0.00	0.44	0.11	0.14	0.31	0.20	0.00	0.21	0.07	0.00	0.10	0.03
**U5**	0.00	0.10	0.05	0.00	0.18	0.06	0.00	0.18	0.09	0.00	0.21	0.11	0.04	0.22	0.11
**U6**	0.00	0.09	0.03	0.00	0.24	0.07	0.00	0.07	0.04	0.00	0.19	0.09	0.00	0.07	0.02
**U7**	0.00	0.03	0.01	0.00	0.02	0.00	0.00	0.05	0.02	0.00	0.05	0.02	0.00	0.00	0.00
**U8**	0.04	0.09	0.06	0.00	0.07	0.04	0.02	0.07	0.05	0.00	0.07	0.04	0.03	0.06	0.05
**U9**	0.02	0.07	0.04	0.00	0.10	0.06	0.00	0.12	0.08	0.00	0.13	0.09	0.00	0.08	0.05
**U10**	0.05	0.09	0.07	0.05	0.11	0.07	0.05	0.13	0.09	0.05	0.12	0.08	0.07	0.19	0.11
**U11**	0.04	0.09	0.07	0.00	0.13	0.06	0.07	0.20	0.11	0.09	0.22	0.16	0.00	0.05	0.01
**U12**	0.00	0.26	0.04	0.00	0.00	0.00	0.00	0.06	0.01	0.00	0.05	0.01	0.00	0.10	0.02
**U13**	0.25	0.47	0.36	0.28	0.65	0.48	0.38	0.71	0.53	0.57	0.85	0.71	0.00	0.28	0.16
**U14**	0.24	0.35	0.30	0.17	0.44	0.30	0.00	0.79	0.41	0.27	0.45	0.32	0.00	0.49	0.32
**U15**	0.00	0.08	0.05	0.04	0.07	0.06	0.00	0.10	0.06	0.04	0.07	0.06	0.00	0.08	0.04
**U16**	0.00	0.03	0.00	0.00	0.00	0.00	0.00	0.04	0.01	0.00	0.11	0.03	0.00	0.00	0.00

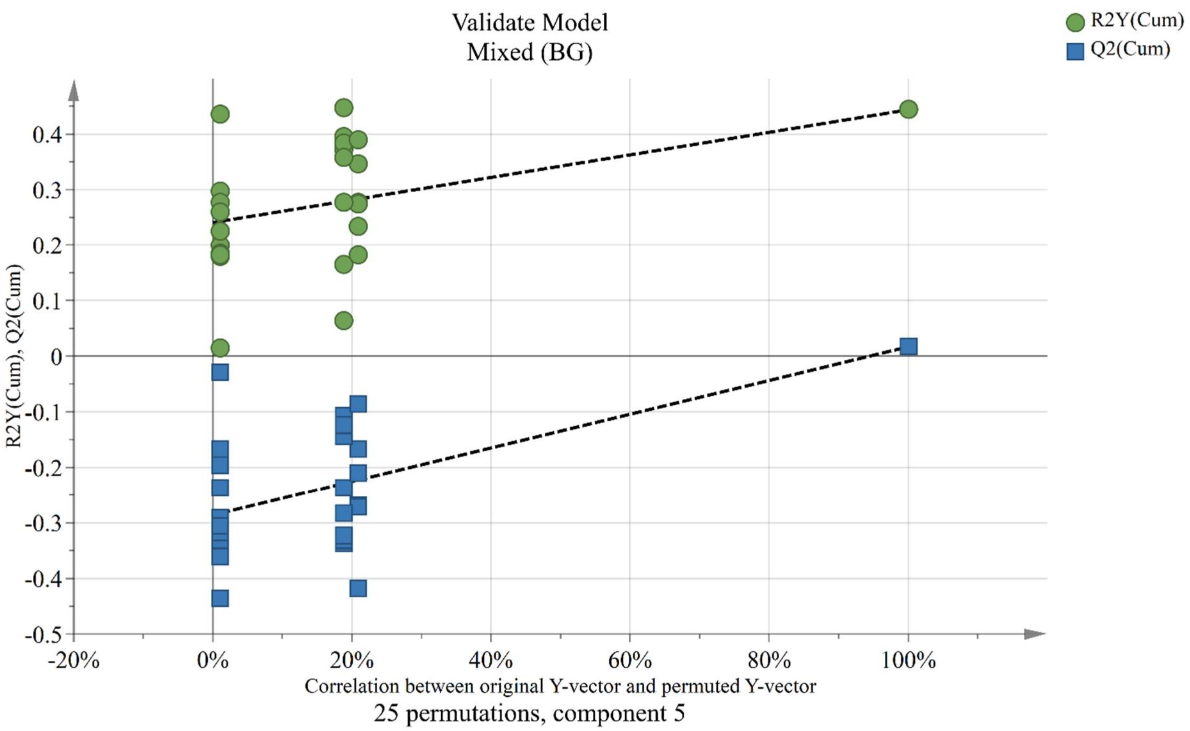

Fig S1. Permutation test for Bulgarian mixed honey class in PLS-DA model

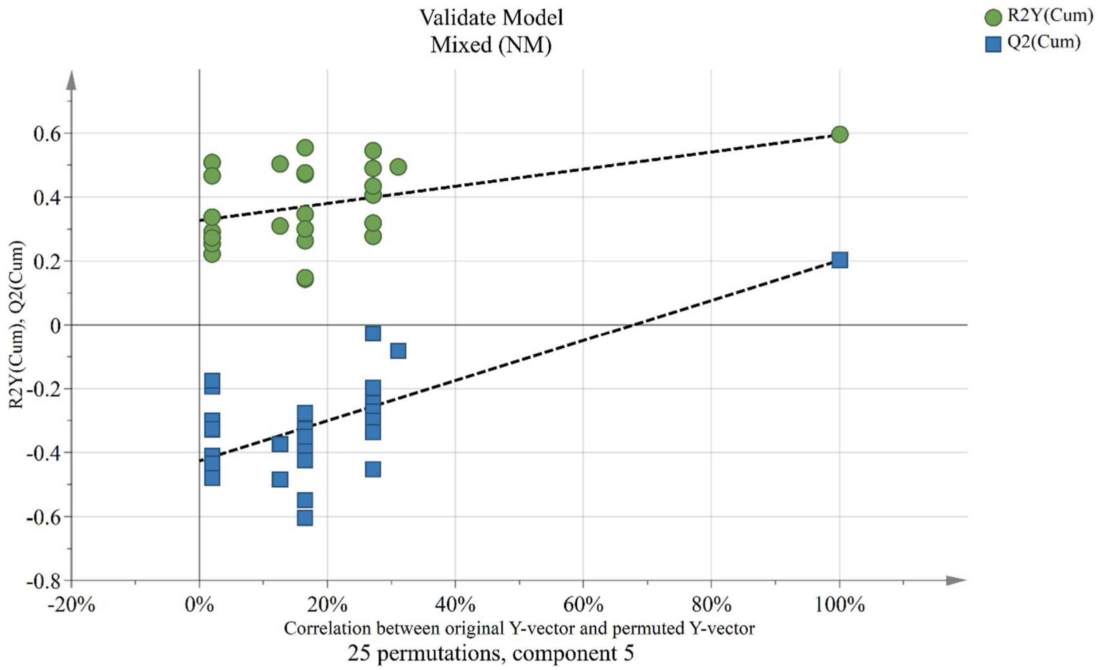

Fig. S2. Permutation test for North Macedonian mixed honey class in PLS-DA model

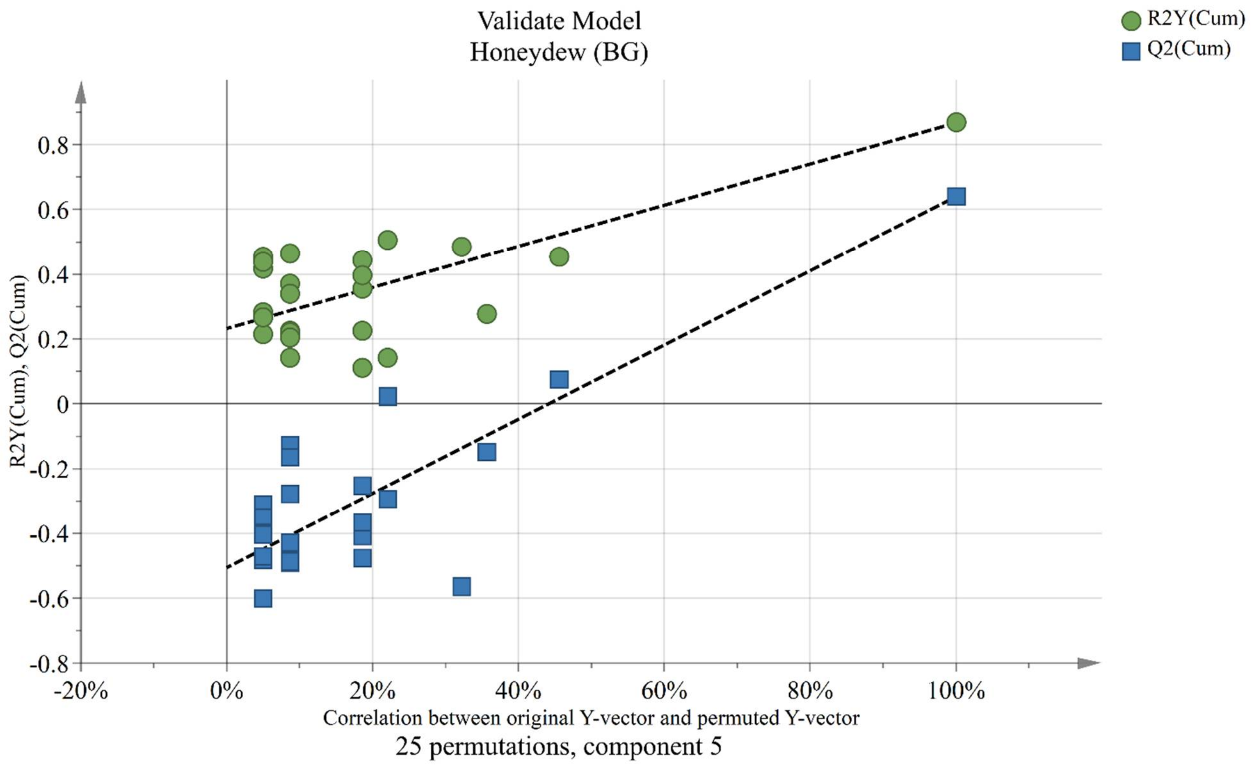

Fig S3. Permutation test for Bulgarian honeydew honey class in PLS-DA model

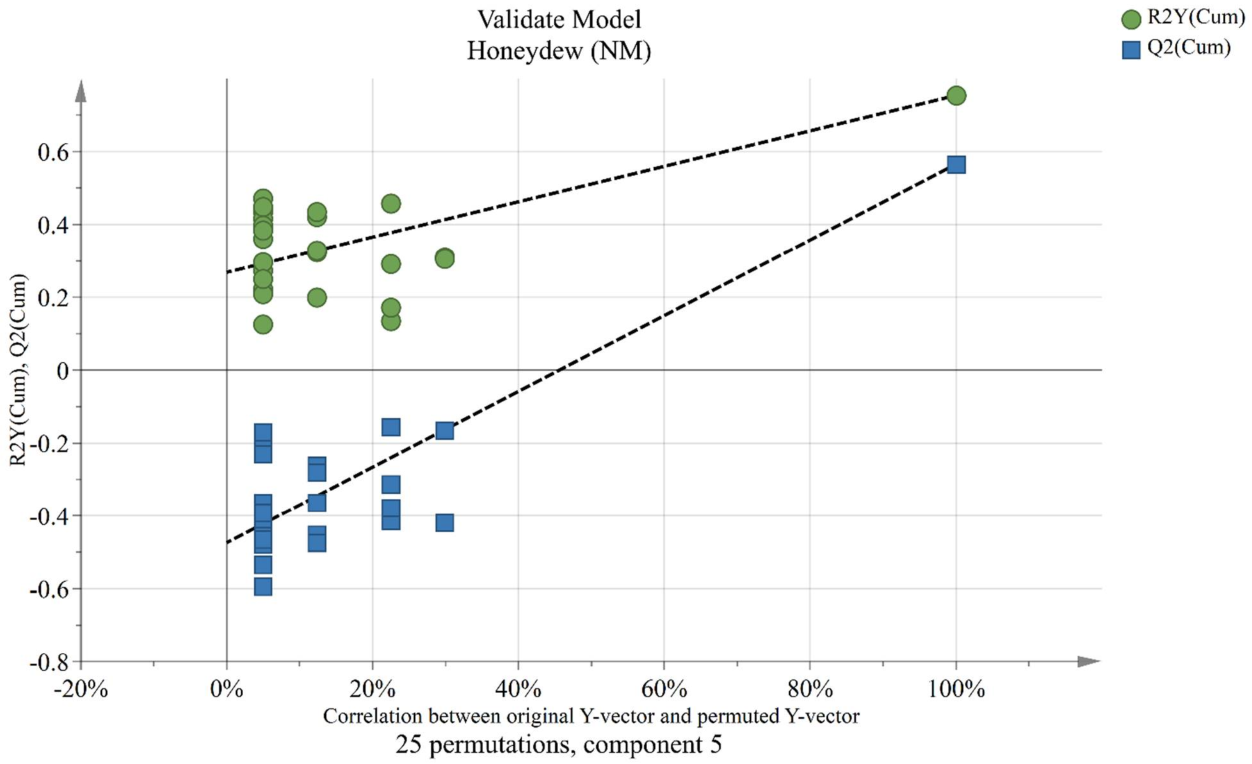

Fig S4. Permutation test for North Macedonian honeydew honey class in PLS-DA model

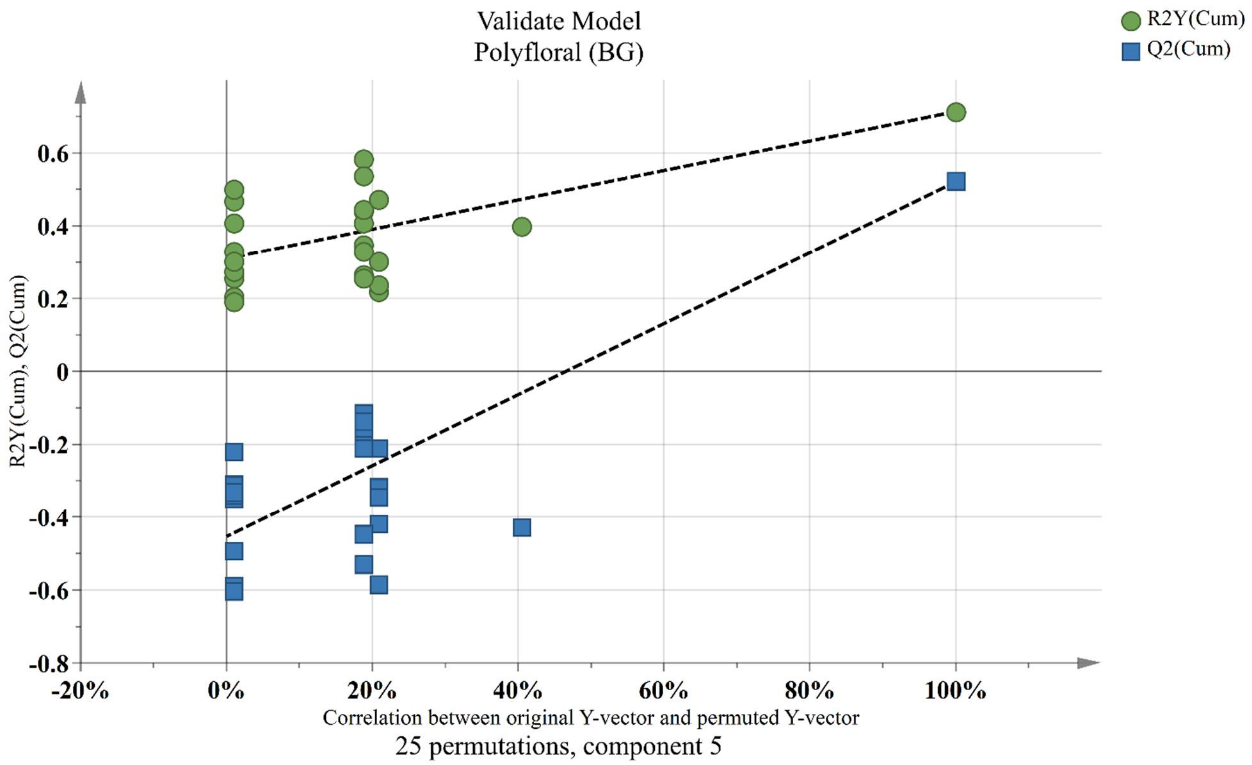

Fig. S5. Permutation test for Bulgarian polyfloral honey class in PLS-DA model

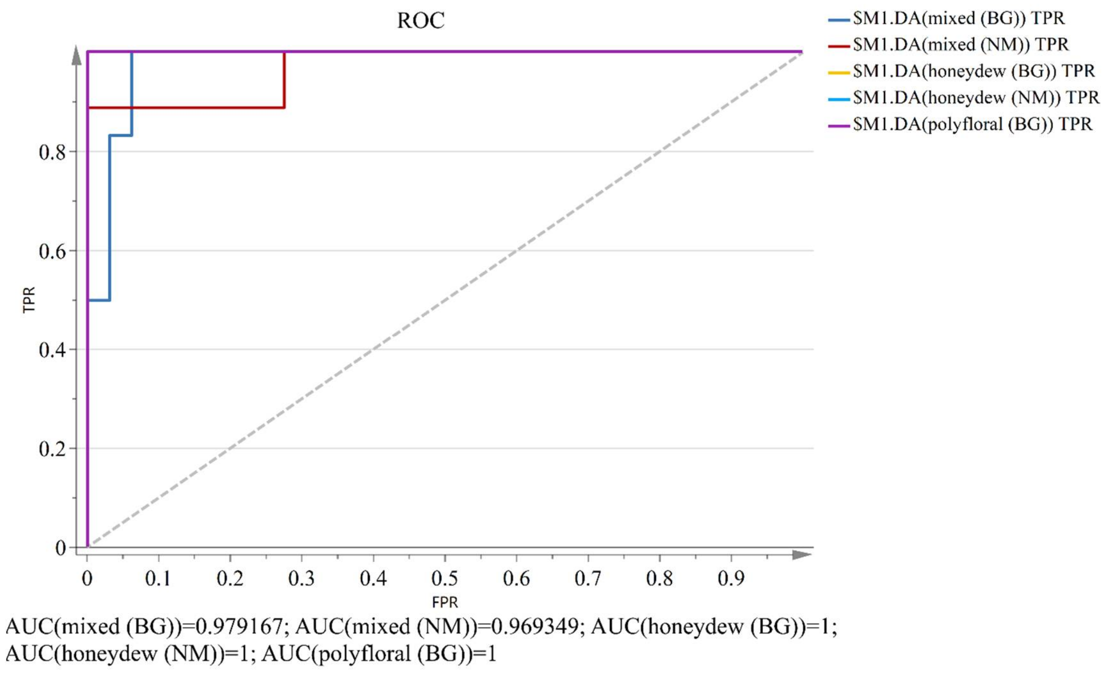

Fig. S6. ROC analysis for Bulgarian mixed, North Macedonian mixed, Bulgarian honeydew, North Macedonian honeydew and Bulgarian polyfloral honeys

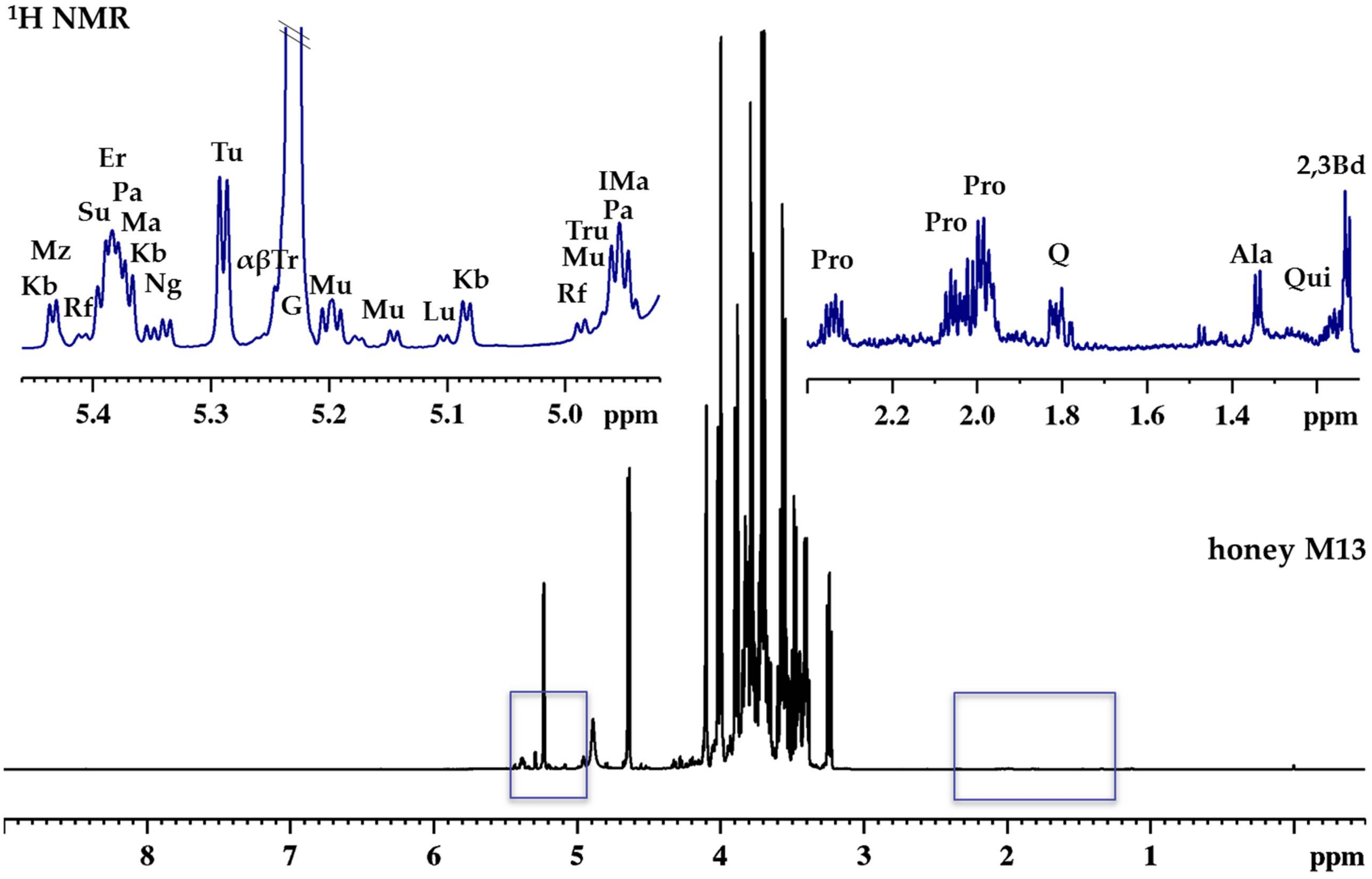

Fig. S7. Full ^{1}H NMR spectrum of sample M13 and assignment of the identified signals in the regions 1.10 – 2.40 ppm and 4.90 – 5.45 ppm

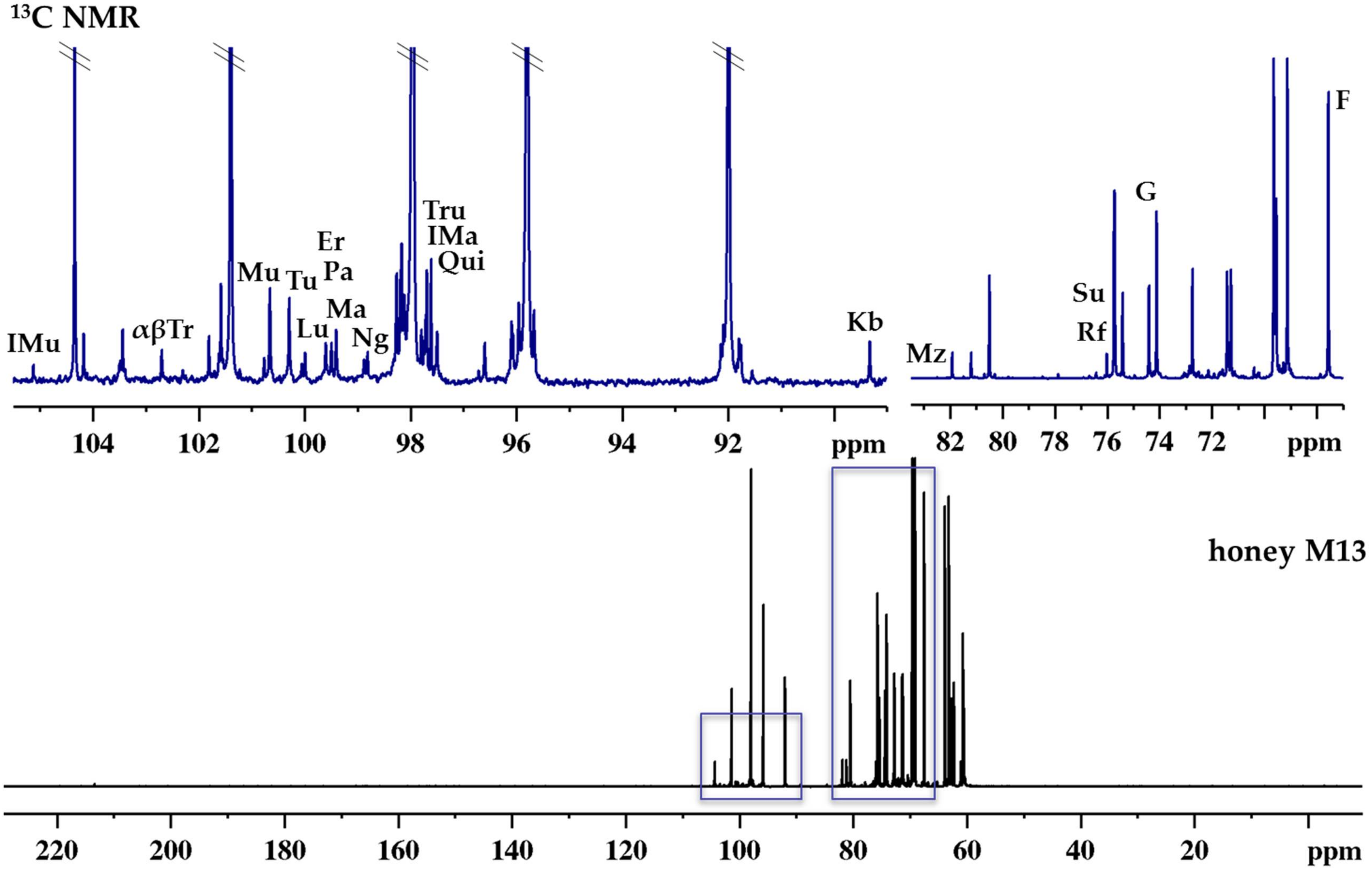

Fig. S8. Full ^{13}C NMR spectrum of sample M13 and assignment of the signals, used in the chemometric analysis shown in Table 1
